# The Role of Occipitotemporal Network for Speed-Reading: An fMRI Study

**DOI:** 10.1007/s12264-024-01251-w

**Published:** 2024-06-27

**Authors:** Dexin Sun, Zhilin Zhang, Naoya Oishi, Qi Dai, Dinh Ha Duy Thuy, Nobuhito Abe, Jun Tachibana, Shintaro Funahashi, Jinglong Wu, Toshiya Murai, Hidenao Fukuyama

**Affiliations:** 1 Research Center for Medical Artificial Intelligence, Shenzhen Institute of Advanced Technology, Chinese Academy of Sciences, Shenzhen, 518055 China; 2 University of Chinese Academy of Sciences, Beijing, 100049 China; 3 Department of Psychiatry, Graduate School of Medicine, Kyoto University, Kyoto, 606-8501 Japan; 4 Medial Innovation Center, Graduate School of Medicine, Kyoto University, Kyoto, 606-8501 Japan; 5 Human Brain Research Center, Graduate School of Medicine, Kyoto University, Kyoto, 606-8501 Japan; 6 Kokoro Research Center, Kyoto University, Kyoto, 606-8501 Japan; 7 Speed Reading Academy, Kyoto, 600-8439 Japan

**Keywords:** Speed reading, fMRI, The occipitotemporal network, Functional connectivity, Effective connectivity

## Abstract

The activity of occipitotemporal regions involved in linguistic reading processes, such as the ventral occipitotemporal cortex (vOT), is believed to exhibit strong interactions during higher-order language processing, specifically in the connectivity between the occipital gyrus and the temporal gyrus. In this study, we utilized functional magnetic resonance imaging (fMRI) with psychophysiological interaction (PPI) and dynamic causal modeling (DCM) to investigate the functional and effective connectivity in the occipitotemporal network during speed reading. We conducted the experiment with native Japanese speakers who underwent and without speed-reading training and subsequently performed established reading tasks at different speeds (slow, medium, and fast) while undergoing 3-Tesla Siemens fMRI. Our activation analyses revealed significant changes in occipital and temporal regions as reading speed increased, indicating functional connectivity within the occipitotemporal network. DCM results further demonstrated more intricate effective connections and high involvement within the occipitotemporal pathway: (1) reading signals originated from the inferior occipital gyrus (iO), distributed to the vOT and the posterior superior temporal sulcus (pSTS), and then gathered in the anterior superior temporal sulcus (aSTS); (2) reading speed loads had modulation effects on the pathways from the aSTS to vOT and from the iO to vOT. These findings highlight the complex connectivity and dynamic interactions within the occipitotemporal network during speed-reading processes.

## Introduction

Reading is the most effective cognitive gateway through which humans acquire new knowledge. Although the average reading speed is ~200–400 words per minute (w/min) for most English-speaking adults, one would like to enhance the speed so as to obtain new knowledge more quickly and efficiently.

Some speed-reading enthusiasts claimed that they can read English sentences with as many as 30,000–40,000 w/min. Multiple strategies may be applied to increase reading speed. One strategy is inhibiting the habit of articulating words and concentrating on keywords or ideas when reading [1]. Another strategy is avoiding sentence rereading [2]. Acquiring a wider eye span through training would also be an effective strategy because the average eye span is usually limited to approximately two to three words [3, 4]. The pacer technique is also available to increase reading speed; a pen or a highlighter can be used as a pacer, and reading speed can be increased by moving the pacer to a faster (yet comfortable) speed [5].

Regardless of the strategy used by each participant, rapid reading requires complicated linguistic processes. Therefore, several brain areas relevant to such processes are thought to be involved in reading speed. In one functional Magnetic Resonance Imaging (fMRI) experiment, wherein participants listened to English nouns presented at various rates ranging from 0 to 130 w/min, increased activation was observed at a rate of 90 w/min in the primary auditory and auditory association areas, but the activity decreased at a rate of 130 w/min [6]. In another fMRI experiment, wherein participants were required to silently read essays at ordinary and rapid speeds, it was shown that neural activation decreased with increased reading speed in the left superior temporal region. Furthermore, other studies have shown increased activation in the occipital areas with an increase in word presentation rate [7–9].

In addition to the brain areas reported in previous studies, areas related to visuospatial, linguistic, and memory functions may be correlated with reading speed. The intraparietal sulcus, precentral sulcus, and cerebellum are relevant brain areas for visuospatial processing [10, 11]. For linguistic-related processing, the superior temporal sulcus (STS), the inferior frontal gyrus, and the ventral occipitotemporal (vOT) areas participate in rapid reading [12–14]. The hippocampus and frontal lobe are relevant brain areas for memory-related processing [15].

To the best of our knowledge, the contemporary reading models of the neural system proposed that visual information input from the inferior occipital (iO) area, and processing in the vOT drives activity in higher-order language areas, such as the posterior superior temporal sulcus (pSTS) and the anterior superior temporal sulcus (aSTS) [16, 17]. However, speed-reading not only results from simple serial processes involving a small number of regions but also from dynamic interactions across multiple brain regions. Several decades after the initial report of this phenomenon, the dynamic interactions across brain regions during speed-reading remain unexplored.

By analyzing fMRI data with advanced analytical methods, the present study examined how the above-mentioned occipitotemporal regions interact during a speed-reading task, when the reading speed of the sentence changes from slow to medium to fast. The high spatial resolution of the fMRI signals makes it suitable for extracting regional interactions (i.e., connectivity). Connectivity analysis is expected to provide more detailed information about brain functions than conventional activation detection approaches [18, 19]. In this study, we show how reading speed affects brain network interactions, and what in their network contributes to achieving speedy reading ability.

## Materials and Methods

### Participants

A total of 23 native Japanese speakers who attended a speed-reading training course at Sokudoku School (https://www.pc-sokudoku.co.jp/) were recruited as participants for this study. Four participants failed to accomplish the entire experiment. Consequently, a total of 19 participants were included in the analysis. Each participant spent 40 min in one training program; each participant took 9–200 training programs (Table 1). These 19 participants (7 females, 12 males), ranging between 20 and 46 years of age (mean age = 32.4 years, SEM = 2.13 years), were right-handed (assessed using a handedness inventory) [20], and had either normal or corrected-to-normal vision. The speed-reading training program at Sokudoku School primarily comprises six courses: (1) Expanding the field of view, involving a series of practices that show how to expand and focus the view during reading; (2) Shifting gaze points, focusing on improving reading speed and efficiency by controlling the movement of the eyes; (3) Word recognition automation, through extensive practice for the quick recognition of characters during reading; (4) Characters imaging, aiming to associate characters with images or create mental associations for memorization and understanding; (5) Association method, involving the use of association techniques to enhance character memory and connect information to existing knowledge points or images; (6) Parallel imaging, including techniques for parallel association, comparing two or more concepts side by side to facilitate deeper understanding. After extensive training in the speed-reading of Japanese texts, these participants were able to read such texts at a speed of 7,030–29,250 characters per minute (c/min; mean speed = 11,280.5 c/min, SEM = 2,214.2 c/min). In general, native Japanese speakers who had never undergone speed-reading training could read Japanese text at a speed of 506–3,610 c/min (mean speed = 1,069.6 c/min, SEM = 173.7 c/min). This difference showed that the selected participants were able to read Japanese text at a significantly faster speed than ordinary Japanese speakers.

**Table 1:** Characteristics of the training group and comparison group

	Training group (*n* = 19) mean (SEM)	Comparison group (*n* = 19) mean (SEM)	*P-*value
Age	32.4 (2.13)	29.8 (1.26)	0.06
Sex (female/male)	7/12	8/11	0.77
Education of years	16 (0.34)	15.5 (0.22)	0.43
Training times	54.8 (12.4)	--	--
Maximum reading speed before training (c/min)	1,069.6 (173.7)	--	--
Maximum reading speed after training (c/min)	11,280.5 (2,214.2)	--	--

c/min, characters per minute.

In addition, 19 untrained participants (8 females, 11 males; mean age = 29.8 years, SEM = 1.26 years) were recruited from the Kyoto University postgraduate students. Prior to the current experiment, we ensured that the participants had no experience regarding formal training in speed reading. As the comparison group, they were assigned identical experimental tasks and questions to the training group. The detailed data and *P*-values for all characteristics are presented in Table 1 for both the training and comparison groups.

All participants provided written and verbal informed consent before the start of the experiment. Prior to entering the MRI scanner, they completed questionnaires at the MRI center and reported their current health conditions and medical histories, including physical injuries and mental disorders. All participants were fully debriefed and received enough payment. The experimental procedures for this study were approved by the Institutional Ethics Committee of Kyoto University and performed in accordance with the Declaration of Helsinki.

### Experimental Stimuli

Experimental texts from diverse authors were selected from Japanese expository essays, encompassing all types of Japanese characters (approximately 53% hiragana, 13% katakana, and 32% kanji). Rigorous selection ensured participants’ unfamiliarity with the texts. We chose three texts with approximately the same total number of characters (around 27,000) in a whole experiment, edited for consistency, each of which was separated into nine 30-s videos. We maintained uniformity in the number of lines (three lines, around 60 characters) per page across the experimental texts. Categorizing videos based on varying presentation speeds (slow speed = 50 pages/min, medium = 100 pages/min, fast speed = 150 pages/min) and each speed condition contained three videos. Therefore, every slow-speed video had 25 pages around 1,500 characters, every medium-speed video had 50 pages around 3,000 characters, and every fast-speed video had 75 pages around 4,500 characters. In the MRI scanner, each video was presented on a screen (visual angle, 11.18° × 10.20°) mounted on the head of the scanner that bore a uniform black background. The participants viewed each video through a mirror on a head coil positioned over their eyes.

### Experimental Paradigm

**Fig. 1:**
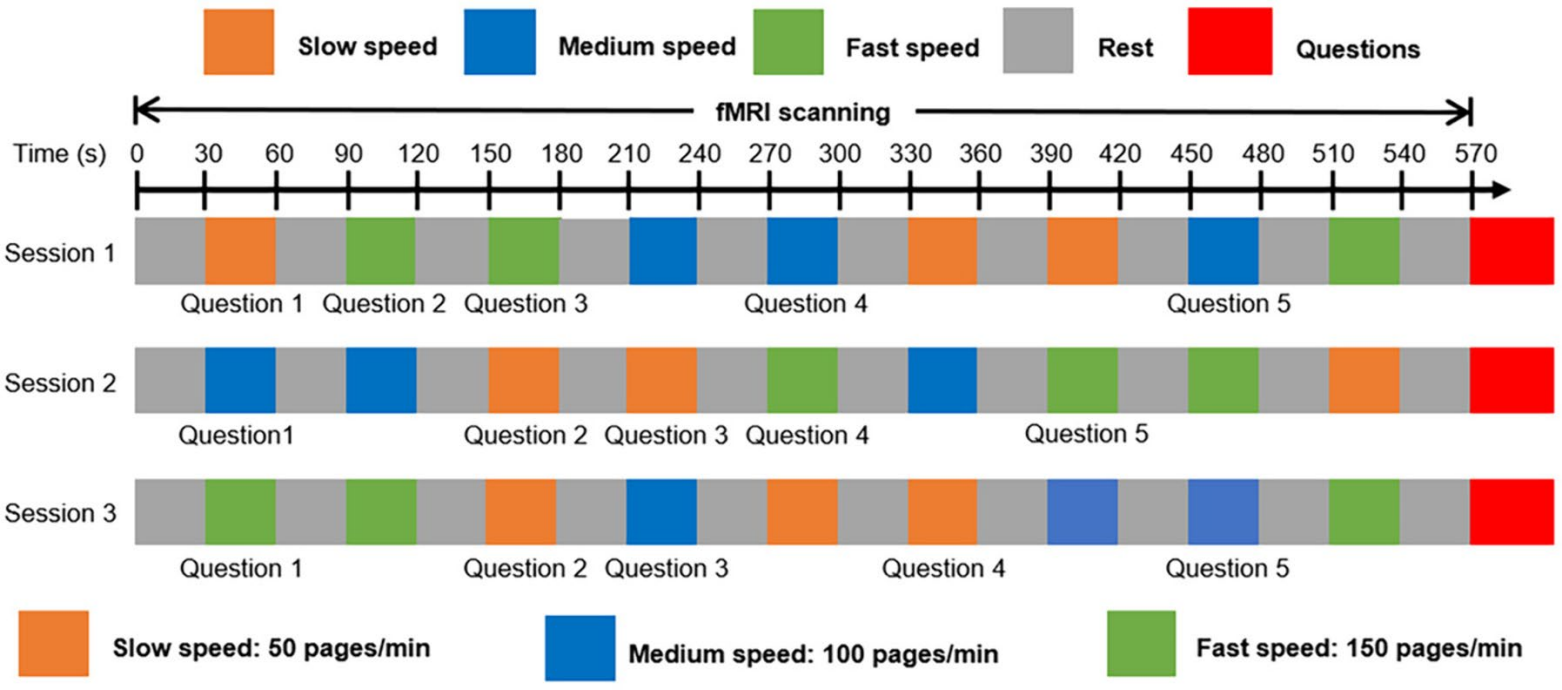
The setup for the fMRI experiments. Three fMRI sessions, each lasting 570 s, were conducted. Each speed condition comprised 9 blocks lasting 30 s, mirroring the duration of the rest blocks. Each session consisted of a total of 9 task blocks and 10 rest blocks. Following each scanning, five pseudo-random questions were presented to assess speed-reading performance.

A block design fMRI experiment utilized 19 blocks per fMRI session, alternating between 9 task blocks and 10 rest blocks (Fig. 1). Each block consisted of a 30-s video. Participants engaged in 3 task blocks for each speed condition (slow, medium, and fast) per session, presented in a pseudo-random order and the same order as reading the text. During rest blocks, participants focused on a fixation cross displayed on the screen. Three sessions, each featuring a whole text including 9 task blocks (9 videos), were recorded for each participant. Throughout the session, participants attended to sentences presented as video during task blocks and fixed their gaze at the fixation cross during rest blocks. After the fMRI scanning, all participants were asked to answer five questions related to the content of the text. They were well-proportionately hidden within the slow, medium, and fast speed blocks, and participants were required to select one option of three by pressing a corresponding button. Owing to the adjustment of the position where the content related to the question appeared, the sequence of five questions was pseudo-randomly arranged for slow, medium, and fast reading speeds. Stimulus presentation and the collection of behavioral responses were controlled using E-Prime software (version 3.0; Psychological Software Tools Inc., Pittsburgh, USA).

### fMRI Data Acquisition

All images were acquired using an MRI machine with a 3-Tesla magnet equipped with a 32-channel phased-array head coil (Verio, Siemens, Munich, Germany) located at the Kokoro Research Center, Kyoto University, Japan. Functional images were obtained using a T2*-weighted gradient echoplanar imaging sequence with the following parameters: echo time (TE)/repetition time (TR), 30 ms/2,500 ms; flip angle, 90°; field of view (FOV), 192 mm × 192 mm; matrix, 64 × 64; 38 interleaved axial slices of 3-mm thickness without gaps; resolution, 3 mm × 3 mm × 3 mm voxels. Structural scans were also acquired using T1-weighted 3-dimensional magnetization-prepared rapid gradient echo sequences (TE, 3.51 ms; TR, 2,000 ms; inversion time, 990 ms; FOV, 256 mm × 256 mm; matrix, 256 × 256; resolution, 1.0 mm × 1.0 mm × 1.0 mm; altogether, 208 total axial sections without gaps).

### Preprocessing of fMRI Data

The acquired imaging data were preprocessed and analyzed using Statistical Parametric Mapping (SPM12) (Wellcome Department of Imaging Neuroscience). Each participant’s Echo Planar Imaging (EPI) images were corrected for geometric distortions caused by susceptibility-induced field inhomogeneity. This was performed using a combined correction for static distortions and changes in distortions caused by head motion. Static distortions were calculated using the FieldMap toolbox to process each participant’s B_0_ field map [21]. All functional brain volumes were realigned to the first volume and spatially normalized to a standard stereotactic space using the template in the Montreal Neurological Institute (MNI) space. These images were resampled into 2 mm × 2 mm × 2 mm voxels during the normalization. All EPI images were smoothed using an 8-mm Gaussian kernel. The data were high pass-filtered with a time constant of 128 s [22].

### Whole-brain Activation Analyses

For the training and comparison groups, every session of fMRI data was modeled on a voxel-by-voxel basis, and three variables in the task condition (slow, medium, and fast speeds) were used as regressors, based on the general linear model (GLM) [23]. Each stimulus onset was modeled as a block design in a condition-specific “speed-reading task” lasting 30 s per block. The resulting stimulus functions were convolved with a canonical hemodynamic response function, which provided the regressors for GLM. Finally, group-level analysis was conducted using a mixed-effects approach, and the independent sample *t*-test was performed across participants to investigate the brain regions involved in the variability of responses in the slow, medium, and fast reading conditions. Additionally, to assess the specificity of results for those with speed-reading training, we investigated the training group > comparison group contrast across three-speed conditions. For illustrative purposes only (i.e., not for statistical inference), all fMRI activations in our work were presented using a voxel-level threshold of *P* <0.05 with false discovery rate (FDR) correction and an extent threshold of >20 voxels [24–26]. The results were inclusively masked with the search mask as described in this article.

### Parametric Modulation Analysis

To assess the activation related to each condition in comparison to the baseline for individual participants, we performed a parametric modulation analysis [27] with 1^st^-order polynomial expansion to investigate the modulation effect of reading speed in each participant. In our work, the events can be modulated by three parameters (slow, medium, and fast speed) using SPM12. In the parametric modulation analysis, for each participant, we first combined the onsets of three-speed conditions into one and set the parametric modulators (condition onsets: [slow medium fast]^T^, parameter matrix values: [−1 0 1]^T^). After model estimating, we could obtain the designed matrix that had two rows for each session. The first row of the designed matrix is the common reading condition and the second row is the modulated matrix by parameters, i.e. the contrast [0 1] of designed matrix represents the speed positive (speed increasing from slow to medium and to fast, and modulated matrix [28] is [0 1]·[−1 0 1;−1 0 1]^T^ = [−1 0 1]^T^), while the contrast [0 −1] represents the speed negative (speed decreasing from fast to medium and to slow, and condition matrix is [0 −1]·[−1 0 1;−1 0 1]^T^ = [1 0 −1]^T^). The time series from each voxel was high-pass filtered (1/128-Hz cut-off) to remove low-frequency noise and signal drift. The motion parameters were included as predictors of no interest in the regression model.

Among these contrasts, we focused on the speed positive and speed negative contrasts. The contrast images from the individual first-level analyses were entered into a second-level random-effects analysis. Different contrast maps were created, and one-sample *t*-tests were used to compare each speed-reading condition with the baseline to assess the overall activity within the whole sample. Statistical analyses of the functional data were performed using a mixed-effects model. Furthermore, we investigated the group difference between speed-negative and speed-positive contrasts between the training group and the comparison group.

### Psychophysiological Interaction Analysis

Psychophysiological interaction (PPI) is a hypothetical method [29] that calculates the degree of effective functional connectivity between one or more predefined brain regions of interest and other brain regions. To examine the functional connectivity among different cortical areas during speed-reading and its modulation depending on the difference in reading speed, we performed PPI analysis using the obtained fMRI data. PPI was recorded across the entire brain, in which regions of the voxel boosted the signal changes associated with seed regions of interest (ROIs) during task performance as well as the degree of change in speed regulation for speed-reading tasks. We used PPI analysis to identify the brain regions that were significantly associated with changes in reading speed in the ROI masks.

Considering the established role in reading, we mainly concentrated on the functional connectivity on the left occipitotemporal network. The time series volume of interest (VOI) was selected based on an effect across groups (the training group > comparison group) for the speed negative contrast. For the PPI analysis, we selected the vOT ([−38, −40, −20], radium 6 mm) as the seed region and extracted the VOI. Next, for each participant, we investigated the speed-contrast effects on PPI. We employed the *t*-test to analyze the functional connectivity within the training group and the comparison group separately. Finally, we examined the differences between the two groups.

### Dynamic Causal Modeling Analysis

Building upon the group differences identified in the PPI analysis, we aimed to ascertain the effective connection using dynamic causal modeling (DCM) analysis. Given that the PPI analysis only unveiled changes in connectivity between the vOT and other related regions in the occipitotemporal pathway, we focused on the experimental stimuli’ effects on the interactions among these regions. The DCM [30] provides a framework that describes the information flow between neurons in various predefined ROIs and accounts for changes in bold responses during task performance. In a DCM, there exist three distinct types of parameters: input sensory parameters, that is, the extent to which brain regions react to experimental stimuli; fixed parameters, which characterize effective connectivity among regions; and modulatory parameters, which delineate alterations in effective connectivity induced by experimental conditions.

From the results of the PPI analysis described above, we selected four ROIs in the left hemisphere with the radius of 6 mm, that is, the inferior occipital gyrus (iO; [−36, −74, −12]), the ventral occipitotemporal cortex (vOT; [−38, −40, −20]), the posterior superior temporal sulcus (pSTS; [−64, −18, 2]), and the anterior superior temporal sulcus (aSTS; [−56, 0, −14]). The iO is a brain region located in the occipital lobe that is primarily responsible for processing visual information and is a part of the visual sensory input. Thus, we set the iO as the sensory input region. The vOT is involved in word recognition. Both the pSTS and aSTS are important for motion perception and information recognition. We speculated that the information flow involving these brain regions might be modulated by the speed of reading.

The first principal component of the time series for each VOI was used for the analyses. We tested possible models to describe the connectivity of regions using fixed-effect Bayesian model selection to select the optimal model. We estimated all DCMs using SPM12, which allowed us to obtain the posterior distribution of the model parameters and the probability of each model. With the initial assumption that each speed condition would independently affect one of the ten connections or simultaneously impact two of them, resulting in a total of 10 + ${\text{C}}_{10 }^{2}$ = 55 models for each speed condition. In total, we constructed 55 DCMs × 3 conditions × 2 groups = 330 DCMs overall. Then, using the slow condition as a baseline, we compared the expected posterior probability (Ep) values of fast and slow speeds, as well as medium and slow speeds. In addition, a group comparison was done for each of the three task speeds.

## Results

### Behavioral Results

In the training group, we observed no significant difference in accuracy among slow speed-reading (mean [SEM], 69.1% [3.5%]), medium speed-reading (mean [SEM], 60.9% [2.9%]), and fast speed-reading (mean [SEM], 63.1% [4.9%]) conditions. This analysis considered training times, and maximum reading speed before and after training as covariates. Furthermore, there was no significant difference in overall accuracy between the training group (mean [SEM], 64.4% [3.8%]) and the comparison group (mean [SEM], 61.4% [1.9%]), after controlling for age and years of education. Crucially, despite the tendency of differences in mean accuracy under different speed conditions, no significant differences were observed. This suggests that participants in both groups were able to read rapidly presented sentences with comparable accuracy to slowly presented sentences. Furthermore, the results confirmed that participants were similarly attentive to the tasks across three conditions.

### Whole-Brain Activation Results

**Fig. 2:**
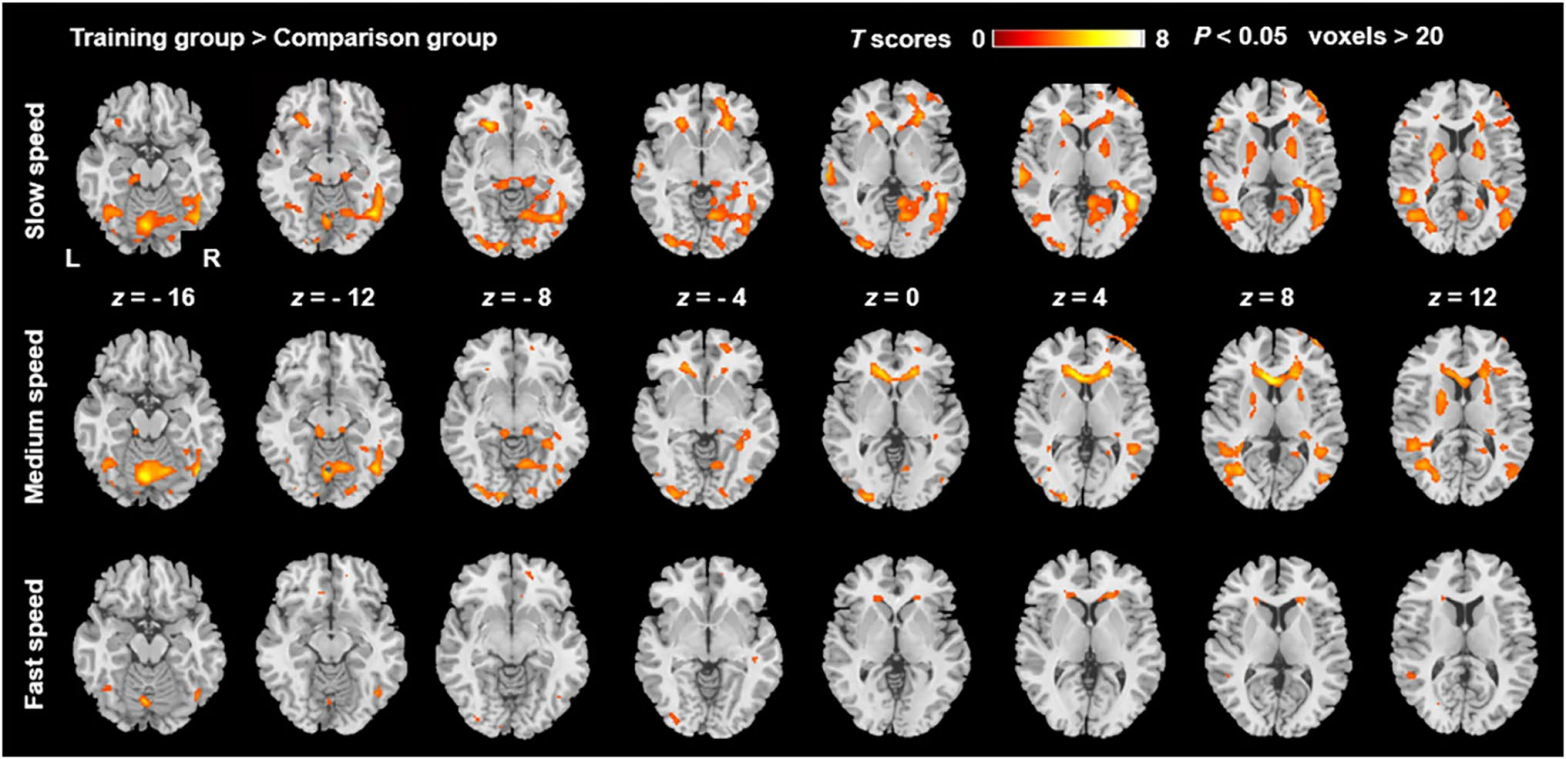
The whole-brain between-group (training group > comparison group) differences activation for each speed condition at *P* < 0.05 (FDR corrected at voxel level) with an extended threshold of > 20 voxels. The color bar indicates *T* scores. L, the left hemisphere; R, the right hemisphere.

We specifically compared the whole-brain activation (Fig. 2) between the training and comparison groups under three different speed conditions (slow, medium, and fast speeds). It was noteworthy that as the speed increased, the difference in brain activation gradually diminished and decreased in the training group > comparison group contrast. Across these three conditions, the regions where the activation was larger in the training group (Fig. 2) included the left STS, the left middle temporal gyrus (MTG), the left and right iO, the left middle occipital gyrus (MOG), the left inferior parietal gyrus (IPG), the left precentral gyrus, and the left calcarine fissure, respectively. No significant difference was found in the training group < comparison group contrast.

### Parametric Modulation Analysis

**Fig. 3:**
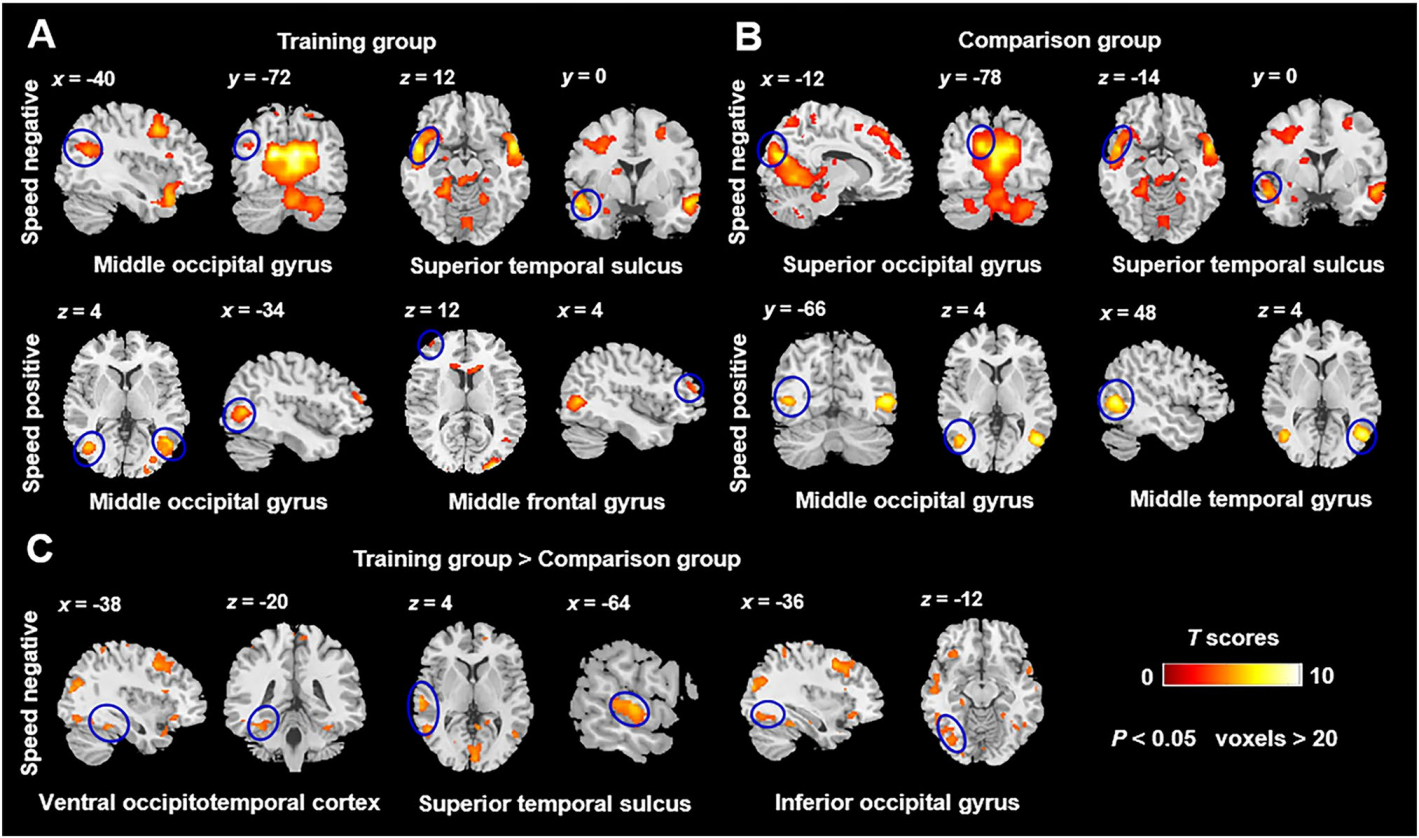
Parametric modulation by changing the reading speed. Speed negative contrast and speed positive contrast for the training group (**A**) and comparison group (**B**). **C** Group differences (training group > comparison group) in the speed negative contrast. The blue circles present the brain regions mentioned below. The color bar indicates *T* scores (voxels > 20, *P* < 0.05, FDR corrected at the voxel level).

Under the speed-negative contrast (Fig. 3A and Table 2) in the training group, significant regions of modulation included the MOG and the STS. In the same contrast, the comparison group (Fig. 3B and Table 2) exhibited significant activation in the STS and the superior occipital gyrus (SOG). Conversely, under the speed-positive contrast, activated regions were relatively sparse; in the training group, they primarily comprised the MOG and the middle frontal gyrus (MFG), while in the comparison group, they included the MOG and the MTG. Furthermore, we investigated the group differences between the training and comparison groups (Fig. 3C and Table 2). The speed negative contrast was found to be higher in the training than the comparison group in the vOT, STS, and iO. This suggests that as reading speed increases, reduced activation of these regions is more notable in the training group. In no region, this contrast was found to be higher in the comparison group than in the training group. Regarding the speed-positive contrast, no significant group differences were observed.

**Table 2:** Coordinates of activation in the occipitotemporal pathway associated under speed negative and speed positive conditions (voxels > 20, *P* < 0.05, FDR corrected at the voxel level)

Peak MNI coordinate regions	Cluster size (voxels)	Peak MNI coordinate			
		*X*	*Y*	*Z*	*T*
*Training group (speed negative)*					
Temporal_Mid_L	2,195	−48	−4	−24	6.89
Temporal_Sup_L	682	−50	4	−14	7.63
Temporal_Pole_Sup_L	588	−50	16	−20	8.33
Occipital_Mid_L	153	−38	−64	24	4.85
*Training group (speed positive)*					
Occipital_Mid_L	81	−44	−68	4	5.94
Occipital_Mid_R	53	30	−94	10	6.38
Frontal_Mid_L	32	−44	48	18	4.28
*Comparison group (speed negative)*					
Temporal_Mid_L	467	−50	−36	4	5.67
Occipital_Sup_L	124	−12	−78	22	3.59
Temporal_Sup_L	106	−46	−36	4	5.17
Temporal_Pole_Sup_L	43	−28	10	−24	4.93
*Comparison group (speed positive)*					
Occipital_Mid_L	97	−44	−66	4	6.27
Temporal_Mid_L	82	−46	−58	4	6.03
*Training > Comparison group (speed negative)*					
Temporal_Mid_L	467	−64	−16	0	4.48
Occipital_Mid_L	359	−36	−84	20	4.39
Temporal_Pole_Sup_L	235	−46	18	−24	5.13
Posterior Temporal_Sup_L	94	−64	−20	2	4.64
Anterior Temporal_Sup_L	51	−54	−4	−14	4.10
Occipital_Inf_L	88	−36	−74	−12	3.97
Ventral Occipitotemporal Cortex	36	−38	−40	−20	3.87

MNI, Montreal Neurological Institute; Mid, middle; Sup, superior; Inf, inferior; L, the left hemisphere; R, the right hemisphere.

### Psychophysiological Interactions

**Fig. 4:**
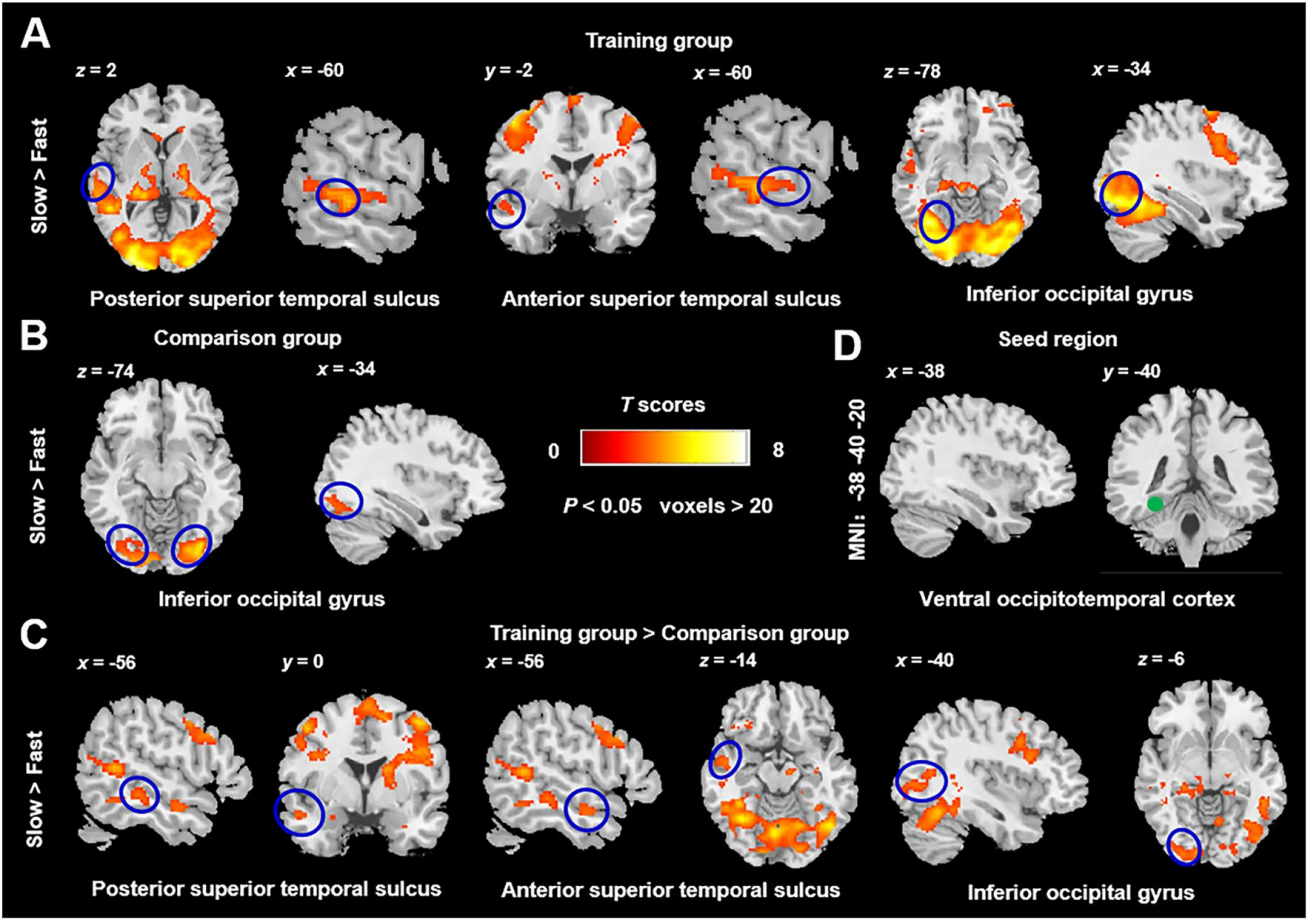
Functional connectivity modulated by reading speed. The training group (A), comparison group (**B**), and between-group differences (**C**; training group > comparison group) in the slow > fast contrast. **D** The seed region (green point) was set at vOT. The blue circles present the brain regions mentioned below. The color bar indicates *T* scores (voxels > 20, *P* < 0.05, FDR corrected at the voxel level). MNI, Montreal Neurological Institute space.

Utilizing the vOT with a radius of 6 mm as the seed region (Fig. 4D), we investigated the patterns of functional connectivity in the left occipitotemporal pathway in response to changes in reading speed. Among the investigated contrasts, i.e., fast > slow, medium > slow, slow > fast, medium > fast, only the contrast of fast > slow demonstrated significant results. Namely, in the training group (Fig. 4A and Table 3), the activation between the vOT and STS, as well as iO, was modulated by the slow > fast contrast. In the comparison group, the vOT and iO are the only regions where the activations were modulated by slow > fast contrast (Fig. 4B and Table 3). Furthermore, the modulation of slow > fast was significantly stronger in the training group compared to the comparison group at the vOT and pSTS, as well as the aSTS and iO regions (Fig. 4C and Table 3). Taken together, the functional connectivities of multiple regions in the occipitotemporal pathway were significantly reduced in the fast reading condition, especially in the training group.

**Table 3:** Coordinates of functional connectivity modulated by the reading speed. The vOT was set as the seed (voxels > 20, *P* < 0.05, FDR corrected at the voxel level)

Peak MNI coordinate regions	Cluster size (voxels)	Peak MNI coordinate			
		*X*	*Y*	*Z*	*T*
*Training Group (slow > fast)*					
Occipital_Inf_L	627	−44	−70	−12	9.91
Temporal_Sup_L	559	−50	−44	12	6.17
Temporal_Pole_Sup_L	339	−54	8	−10	4.78
Comparison group (slow > fast)					
Occipital_Mid_L	454	−14	−90	−4	5.94
Occipital_Inf_L	301	−36	−90	−12	6.01
Occipital_Inf_R	208	30	−84	−10	7.58
*Training Group > Comparison group (slow > fast)*					
Occipital_Mid_L	275	−28	−76	14	4.48
Posterior Temporal_Sup_L	31	−64	−18	2	2.98
Anterior Temporal_Sup_L	33	−56	0	−14	3.10
Occipital_Inf_L	184	−36	−74	−12	4.53

MNI, Montreal Neurological Institute; Mid, middle; Sup, superior; Inf, inferior; L, the left hemisphere; R, the right hemisphere.

### Dynamic Causal Modeling Analysis

**Fig. 5:**
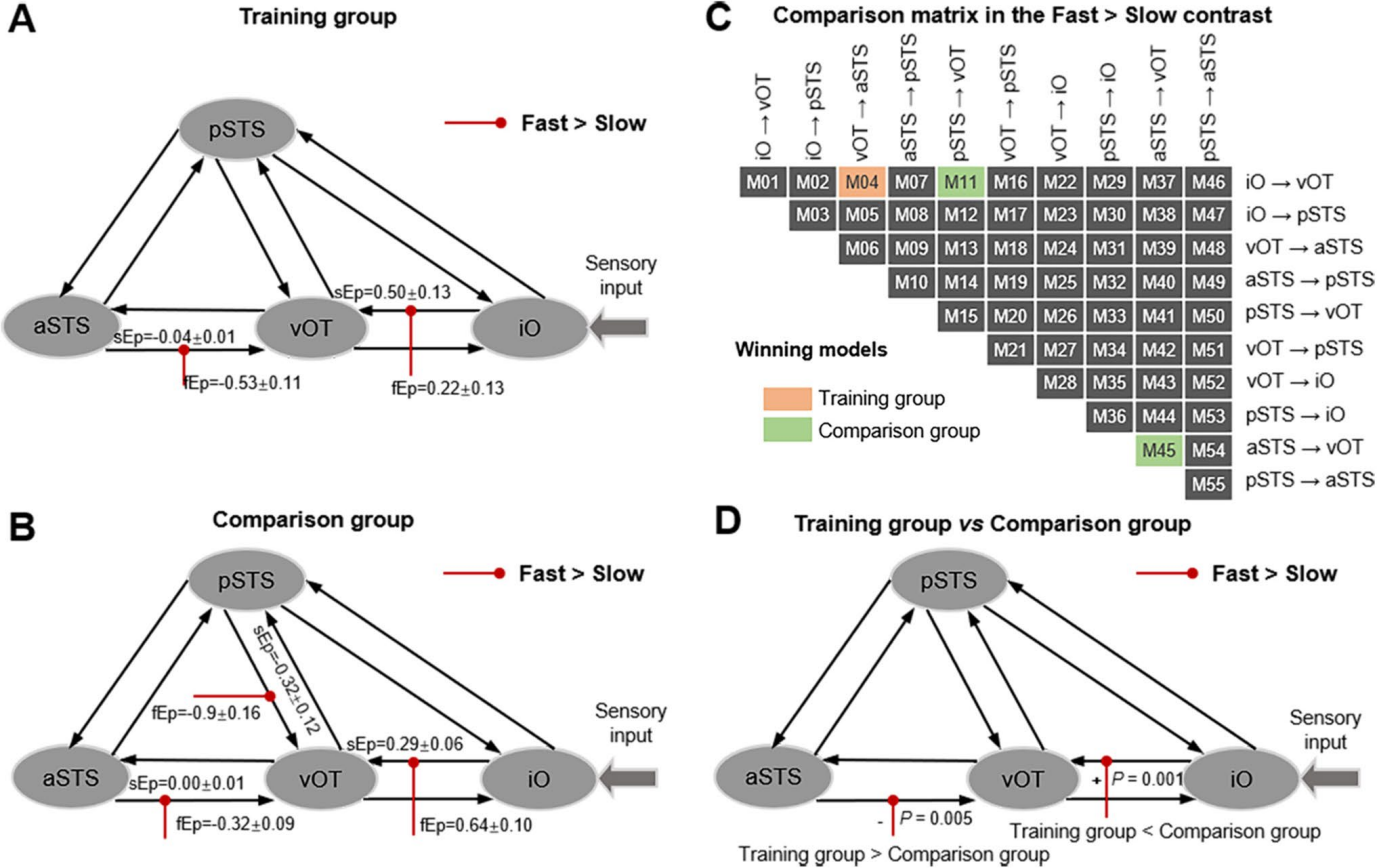
Effective connectivity in the occipitotemporal network and the Bayesian model comparison matrix. sEp, Ep values of slow speed modulated effect; fEp, Ep values of fast speed modulated effect. For the training group (**A**) and comparison group (**B**), sEp and fEp (mean ± SEM) of the modulation connectivity (red line) in the fast > slow contrast. **C** Bayesian model comparison matrix for training and comparison groups. The abscissa and ordinate of the matrix represent the modulation connection of the speed effect. M04 in the training group, and M11 and M45 in the comparison group have the highest exceedance probability. **D** The positive (+) and negative (−) difference of modulatory connection for the training and comparison groups, respectively.

The Bayesian model comparison matrix (Fig. 5C) illustrated 55 models under slow and fast speed conditions, with the abscissa and ordinate representing directional connections. The models with the highest exceedance probability were highlighted for the training group (orange, M04) and for the comparison group (green, M11 and M45).

Fig. 5 displayed the Ep values of the slow speed modulated effect (sEp) and fast speed modulated effect (fEp), and red lines with significant (*P* <0.05) modulation connection of the fast > slow effect from the source region to the target region. Fig. 5A (as well as M04 in Fig. 5C) demonstrated the modulation of reading speed on directional connectivity was inhibitory from aSTS to vOT (sEp ± SEM = −0.04 ± 0.01; fEp ± SEM = −0.53 ± 0.11), and excitatory from iO to vOT (sEp ± SEM = 0.50 ± 0.13; fEp ± SEM = 0.22 ± 0.13). Fig. 5B (as well as M11 and M45 of Fig. 5C) demonstrated the modulation of reading speed on directional connectivity was inhibitory from aSTS to vOT (sEp ± SEM = 0.00 ± 0.01; fEp ± SEM = −0.32 ± 0.09) and pSTS to vOT (sEp ± SEM = −0.32 ± 0.12; fEp ± SEM = −0.9 ± 0.16), while positive from iO to vOT (sEp ± SEM = 0.29 ± 0.06 and fEp ± SEM = 0.64 ± 0.10).

Finally, we compared the Ep of the Bayesian winning models between the training and comparison groups and obtained effective connections (Fig. 5D) with a significant difference (*P* <0.05) modulated by speed effects. The training group exhibited weaker positive activation (*P* = 0.001) than the comparison group in the connection from iO to vOT in the fast > slow contrast. Conversely, the training group exhibited greater inhibitory activation (*P* = 0.005) in the connection from aSTS to vOT. Generally, with the increasing speed as the modulatory factor, the training group exhibited a more pronounced inhibitory connection from aSTS to vOT, together with a slightly enhanced connection from iO to vOT compared to the comparison group.

## Discussion

Our analysis explored speed-reading training-induced connectivity modulation within a linguistic reading network. We observed changes distributed across the occipitotemporal network at various reading speeds. We identified decreased regional sensitivity from slow to fast reading speeds bilaterally in the temporal and occipital regions. As expected, between-region connections modified by reading speed were predominant in the left hemisphere. Moreover, stronger connections were observed in the feedforward direction from the pSTS to the aSTS and from the left vOT to the aSTS. After reviewing the findings, it can be concluded that speed-reading training primarily affected the connectivity in the left occipitotemporal network.

Although our behavior results showed that the mean accuracy of speed-reading of the training group (64.4%) was higher than that of the comparison group (61.4%), the *P-*values failed to reach statistical significance. In each session, participants must remember the contents of task blocks since the scanning began to answer questions when an MRI scan was finished. Besides, the environmental noise during fMRI scanning is also a potential influence on behavioral measurements. On the other hand, kanji contains more contextual information making it important for Japanese to understand the text rather than hiragana and katakana [31, 32]. Participants could extract answers by primarily focusing on kanji, which reduced the comprehension difficulty. Therefore, individual memory ability, environmental noise, and the features of Japanese characters might be the possible factors attributed to the insignificant difference in behavioral results. Further research with a larger sample size should be warranted to elucidate these findings, particularly when participants are required to answer questions without leaving the MRI scanning room.

### Inhibited Activation in the Occipitotemporal Network

From the results of the whole-brain analysis (Fig. 2), we found that the activated regions were concentrated in the occipital gyrus, temporal gyrus, parietal lobule, and pulvinar thalamus, which are thought to be involved in visual and language processing. For example, a previous study found that the pulvinar thalamus has widespread connections with early visual cortical areas and is important for the regulation of visual attention [33]. Another study indicated that damage to the lower parietal lobe can lead to writing and language barriers and has a strong relationship with text reading [34]. Thus, the results suggest that participants primarily utilized language- and visual-related regions when engaging in speed-reading tasks.

As reading speed increased, our findings (Fig. 3 and Table 2) indicated a robust deactivation of related regions in the occipitotemporal pathway. Increasing the reading speed (from slow to medium or fast) led to greater cognitive loads on the brain, it required dynamic spatial processing as a higher-level cognitive function than reading at normal speed. As reading speed increased, the vOT, STS, and iO showed more inhibited activation in the training group compared to the comparison group (Fig. 3C). These areas were found to play a crucial role in high-level visual processing and semantic comprehension [35, 36]. In the fast-reading condition of our experiment, 2.5 pages (60 characters per page) were presented every 1 s, suggesting that our experimental condition was similar to that known as rapid serial visual presentation (RSVP). It has been shown that the RSVP causes inhibitory effects on visual information processing, especially in the recognition of visual information [37, 38]. The presentation of visual stimuli with shorter intervals in the present experiment may lead to the suppression of related neural representations, especially in the visual areas. However, behavioral results showed no significant difference in accuracy among the three speed-reading conditions in both training and comparison groups, suggesting that rapid serial presentation of text pages had a non-significant effect on visual recognition. Therefore, a stronger inhibitory effect as reading speed increases may not be caused by the rapid serial presentation of text pages in the current experiment.

### Functional Connectivity in the Occipitotemporal Network

To establish the importance of functional connectivity in the occipitotemporal network, we specifically examined the vOT as the seed region (Fig. 4D). As speed increased (Table 3), we observed that the vOT showed a weakening activation between the iO, pSTS, and aSTS in our experiment. A functional neuroimaging study [39] identified the left vOT as a critical component of the visual word form area, extending from the occipital to the temporal lobes, which plays a crucial role in reading comprehension. The pathway from the occipital to the temporal lobes or temporal lobes to the occipital lobes *via* this area is essential for reading comprehension. In addition, our finding is similar to that of previous studies [40] in which reduced linguistic processes were observed as the visual word-switching speed of participants increased. A framework [41] for eye movement control is provided in reading models, encompassing factors such as saccades, word length, and word frequency. In another study [42], it was found that high-frequency words produced less left vOT activation than low-frequency words. This phenomenon was attributed to the limited context comprehension of low-frequency words, leading to longer fixation time and the stronger involvement of vOT in context understanding [43]. In our study, as reading speed increased from slow to fast, the saccade speed of participants was compelled to change, resulting in reduced fixation time available for engaging vOT. Consequently, the increase in speed led to decreased activation in brain areas associated with vOT (Fig. 4). Furthermore, the training group exhibited greater familiarity with the fast reading practices than the comparison group. When the number of characters read within a short period increased (as speed increased), the training group was more adept at swiftly decreasing the fixation time on each character. It is also one possible reason why the left vOT in the training group exhibited larger inhibited activation compared to the comparison group as the reading speed increased (Fig. 3C).

### Effective Connectivity in the Occipitotemporal Network

To investigate the effective connectivity of the occipitotemporal network, we selected the iO, vOT, aSTS, and pSTS to establish the DCMs (Fig. 5C), wherein reading information is the sensory input from the iO through the ventral occipitotemporal cortex to aSTS and pSTS. We observed that with increasing speed, the signal from aSTS to vOT was more inhibited, while the signal from iO to vOT was less enhanced in the training group compared to the comparison group. This observation suggests the involvement of the occipitotemporal pathway, whereby the signal from the occipital lobe is transmitted more heavily to the temporal lobe. We hypothesized that there would be an increased demand in areas of the temporal lobe related to language processing, potentially having various directional pathways bypassing the occipitotemporal network.

According to some anatomical studies [16], there are different pathways for signals from the iO to the aSTS: one is the ventral pathway through the vOT and the other is the dorsal pathway through the pSTS. Reading involves the ventral stream extending from the ventral cortex to the occipitotemporal region along the surface of the temporal lobe. In a previous study [44], the vOT was thought to be involved in the posterior reading circuit, focusing on semantic processing and word integration. The vOT contains several cortical regions dedicated to the higher-level visual processing of scenes, faces, objects, and words, which follow a well-characterized medial-to-lateral topography with regard to their preferred tuning. The reading-related Wernicke’s Area [45] is located in the pSTS of the brain. Therefore, the ventral pathway was a crucial information transmission pathway in our experiment, which participated in higher-level language processing [46], including rapid semantic and lexical integration, as well as text comprehension. Differently, the dorsal stream [47], extending from the dorsal aspect of the occipital lobe to the superior temporal gyrus, is more focused on visual location and semantic integration; there is also an interaction between the dorsal and ventral streams [48, 49]. Our experiment indicates that both the ventral and dorsal pathways are jointly involved in visual attention and the rapid switching of words in space.

Furthermore, STS has always been considered related to written language in some studies. An STS contains various subregions with different functions. One study found [50] that the aSTS in the human brain has a functional preference for processing dynamic facial motion similar to that of monkeys. In another experiment on attention and action observation, the pSTS was found to be sensitive to explicit signals indicating behavior, indicating that the pSTS was more active during joint attention tasks involving more than simple visual processing of action [51]. For the training group compared to the comparison group, the DCM results (Fig. 5D) reveal that as reading speed accelerates, there is a more pronounced inhibitory activation modulation from aSTS to iO and a weaker facilitatory modulation from iO to vOT. A potential explanation is that participants in the training group possess greater experience in speed reading exercises compared to those in the comparison group. This increased experience may enable their brains to transition more swiftly into a state conducive to speed reading, thereby mitigating the excessive cognitive load associated with accelerated reading. Overall, our study established a directional occipitotemporal network for reading in speed loads (medium and fast conditions). This network and connection flow provided a more precise mapping of changes in brain function during our speed-reading experiments.

## Conclusion

In summary, this study showed that speed-reading training is associated with distributed changes across the occipitotemporal linguistic reading network. We identified a mixture of the following: first, during the reading experiment, there was activation of regions responsible for visual processing and reading; in particular, increasing reading speed led to the deactivation of the left occipitotemporal network. Second, we observed that an increase in speed generated inhibition in functional connectivity between the vOT and related regions in the left occipitotemporal pathway. Third, using the iO as the sensory input, DCM results revealed effective connections in the occipitotemporal pathway. Evidence of functional and effective connectivity is essential for understanding how and where linguistic reading processes modulate or steer voluntary speed-reading behaviors.
